# Information theoretic analysis of proprioceptive encoding during finger flexion in the monkey sensorimotor system

**DOI:** 10.1152/jn.00178.2014

**Published:** 2014-10-08

**Authors:** Claire L. Witham, Stuart N. Baker

**Affiliations:** Institute of Neuroscience, Newcastle University, Newcastle upon Tyne, United Kingdom

**Keywords:** proprioception, rate coding, temporal coding

## Abstract

There is considerable debate over whether the brain codes information using neural firing rate or the fine-grained structure of spike timing. We investigated this issue in spike discharge recorded from single units in the sensorimotor cortex, deep cerebellar nuclei, and dorsal root ganglia in macaque monkeys trained to perform a finger flexion task. The task required flexion to four different displacements against two opposing torques; the eight possible conditions were randomly interleaved. We used information theory to assess coding of task condition in spike rate, discharge irregularity, and spectral power in the 15- to 25-Hz band during the period of steady holding. All three measures coded task information in all areas tested. Information coding was most often independent between irregularity and 15–25 Hz power (60% of units), moderately redundant between spike rate and irregularity (56% of units redundant), and highly redundant between spike rate and power (93%). Most simultaneously recorded unit pairs coded using the same measure independently (86%). Knowledge of two measures often provided extra information about task, compared with knowledge of only one alone. We conclude that sensorimotor systems use both rate and temporal codes to represent information about a finger movement task. As well as offering insights into neural coding, this work suggests that incorporating spike irregularity into algorithms used for brain-machine interfaces could improve decoding accuracy.

a key controversy in neuroscience is the extent to which neurons use a rate-based or temporal code (Shadlen and Newsome 1998; Softky and Koch 1993). Many studies have shown that temporal coding can carry more information than average firing rate, but it is unclear how well downstream cells can use any temporal information. More recently, it has been suggested that the brain is able to make use of multiple codes multiplexed across different time scales (see review by Panzeri et al. 2010).

Work on temporal coding has often concentrated on a latency code in response to a stimulus, which requires an external temporal reference to mark stimulus onset (Gawne et al. 1996; Panzeri et al. 2001). The brain, however, does not have fiducial access to the external reference, rendering any stimulus decoding based on latency impractical. Recent attention has switched to candidate codes which generate their own internal temporal references (Panzeri and Diamond 2010). These could include population responses where synchronous activity between neurons is important (Hatsopoulos et al. 1998), the phase relationship between spikes and local oscillations (Huxter et al. 2008; Kayser et al. 2009), and patterns within the firing of single neurons (interspike intervals, ISIs).

Rate and temporal coding take fundamentally opposing views of the variability of ISIs. From the perspective of rate coding, fluctuations represent noise, which can only be overcome by averaging sufficient intervals. Smaller variability thus reflects better encoding of the signal (Shadlen and Newsome 1998). By contrast, temporal coding sees interval variation as the coding space (Softky and Koch 1993); relatively constant intervals then imply a reduced range of signaling possibilities (channel capacity; Rieke 1997).

One practical problem in distinguishing between rate and temporal coding is that these two distinct methods of representation are expected to yield experimental recordings that exhibit properties of both codes. Thus, in a system based purely on rate coding, intervals will fluctuate as the underlying rate modulates to convey signal. Equally, temporal coding will result in changes in firing rate, representing the low-pass-filtered version of the wide-band temporal code. In some situations, it may not be possible to deduce the underlying code from experimentally recorded data (Marom et al. 2009). One feasible approach might be to make measures of spike timing variability that exclude the slow modulation of firing rate. On a rate coding view, these will contain only noise and hence will not code useful information. By contrast, if precise spike timing can be used as a neural code, such “rate-corrected” variability measures should convey additional information.

A variety of metrics have been used in the past to quantify ISI variability. The simplest is the coefficient of variation (CV), which is the standard deviation of the ISIs divided by their mean. This can be related to the Fano factor of the spike count distribution, which is the count variance in a time window divided by the mean and which will equal CV^2^ for a stationary renewal process (Nawrot et al. 2008; Pipa et al. 2013). For a constant-rate Poisson process, the CV and Fano factor equal one. However, CV mixes spike irregularity with the additional variation caused by slow modulation of firing rate, frequently leading to large values of CV in experimental data. Alternative approaches use the entropy of the ISI distribution, which quantifies all aspects of the distribution spread, rather than purely the second moment as measured by CV (Bhumbra and Dyball 2004). As for CV, there is no straightforward way to correct ISI entropy for rate fluctuations. Two measures have been introduced to measure ISI variability independent of rate changes, termed *L*_V_ (local variation of ISI; Shinomoto et al. 2005) and IR (irregularity metric; Davies et al. 2006). Spike irregularity modulates in a task-dependent way in motor cortical neurons (Davies et al. 2006), but this does not provide unequivocal evidence for temporal coding. Irregularity could simply modulate in tandem with rate but provide no additional coding capacity.

Alternative approaches to measure temporally patterned activity rely on the existence of network oscillations, which generate an overt signature as rhythmic modulation of local field potential. An extensive literature has demonstrated that these oscillations modulate with behavioral task or stimulus configuration (Baker et al. 2001; Kilner et al. 2000; Kristeva et al. 2007; Rickert et al. 2005; Riddle and Baker 2006; Rubino et al. 2006; Witham and Baker 2012), although to date we still cannot exclude the possibility that these modulations are simply linked to rate changes and are an epiphenomenon (by-product) of a functional neural code which does not rely on oscillatory activity.

In this study, we have simultaneously measured firing rate, oscillations, and spike irregularity from neural populations in primate sensorimotor cortex, deep cerebellar nuclei, and peripheral afferents. The animals were trained to perform an index finger flexion task that incorporated a 2-s-long holding phase, during which firing rate was relatively stationary compared with the extensive modulations typically seen during movement. Digit displacement and applied force were systematically varied. Using an information theory approach, we first demonstrate that each metric of cell discharge can code information about these task parameters. We then show that measures based on the temporal structure of spike activity often code additional information over and above rate, in favor of a temporal coding hypothesis.

## METHODS

### 

#### Behavioral task.

Two female rhesus macaques (*Macaca mulatta*) were trained to perform a finger flexion task for food reward. The index finger was inserted into a narrow tube, which splinted the finger and constrained movement to the metacarpophalangeal (MCP) joint. The tube was mounted on a lever that rotated on an axis aligned to the MCP joint. Lever movement was sensed by an optical encoder, and a motor exerted torque in a direction to oppose flexion. The task required movement into target (6°, 12°, 18°, or 24° flexion) and holding for 2 s. The lever position had to be maintained within ±1° for the 6° target, ±2° for the 12° target, ±3° for the 18° target, and ±4° for the 24° target for the duration of the hold. The motor was activated to behave like a spring, with an initial torque required to move the lever from its end stop of 48 mN·m and the spring constant chosen in each condition so that the torque required at the target displacement was either 64 or 128 mN·m. The four displacements combined with two torques yielded an eight-condition task; different conditions were presented in a pseudorandom order. At the end of the hold phase, motor torque increased, and the animal released the lever to complete the trial. Food rewards were given randomly every one to five successful trials on a variable ratio scheme. Target and lever displacement were indicated on a display screen viewed by the monkey. The gain of the relationship between screen cursors and actual lever displacement changed on each trial such that the target always appeared in the same position on the screen regardless of the desired finger displacement. This ensured that there was no visual information about the absolute finger displacement.

#### Surgical preparation.

Following behavioral training, each monkey was implanted under general anesthesia and aseptic conditions with a headpiece (to allow head fixation) and a recording chamber placed over the central sulcus (Baker et al. 1999; Lemon 1984). The anesthesia consisted of 3.0–5.0% sevoflurane inhalation in 100% O_2_ supplemented with a continuous intravenous infusion of alfentanil (0.025 mg·kg^−1^·h^−1^). A full program of postoperative analgesia (10 μg/kg buprenorphine, Vetergesic; Reckitt and Colman Products; 5 mg/kg carprofen, Rimadyl, Pfizer) and antibiotic care (10 mg/kg cefalexin, Ceporex; Schering-Plough Animal Health; or 15 mg/kg amoxycillin, Clamoxyl LA; Pfizer) followed surgery. In a further surgery a chamber was implanted, centered over posterior (P) 8.5, mediolateral (ML) 4, to allow access to the deep cerebellar nuclei (DCN). Finally, a spinal chamber was implanted, which allowed access to the C7–C8 dorsal root ganglia (DRG) (Flament et al. 1992). All procedures were carried out under the authority of licenses issued by the UK Home Office under the Animals (Scientific Procedures) Act 1986.

#### Recording.

In daily experiments, a 16-channel microdrive, loaded with either glass-insulated platinum electrodes (M1) or tetrodes (area 3a, area 3b, area 1, area 2, area 5, and DCN), was used to record single units and local field potentials (LFPs) (Eckhorn and Thomas 1993). For the DCN, six sharpened guide tubes penetrated through dura and parietal cortex as far as the tentorium to avoid electrode deviation (Soteropoulos and Baker 2006). For the DRG, a five-channel microdrive loaded with tetrodes was used. Spike waveforms (300 Hz–10 kHz bandpass) were sampled continuously at 25 kHz and saved to hard disk together with lever position and task behavioral markers. Spike occurrence times were discriminated offline using custom-written cluster cutting software (Getspike; Baker SN). Only clean single units with consistent wave shapes and no ISIs <1 ms were used for subsequent analysis.

The different brain areas were identified by a clinical examination of unit receptive fields and by noting the motor responses to microstimulation (13–18 biphasic pulses, 300 Hz, 0.2 ms per pulse, currents up to 60 μA). Some of the data set used in this article contributed to our previous publications (Witham and Baker 2007, 2012; Witham et al. 2010, 2011).

#### Analysis.

**Fig. 1.**
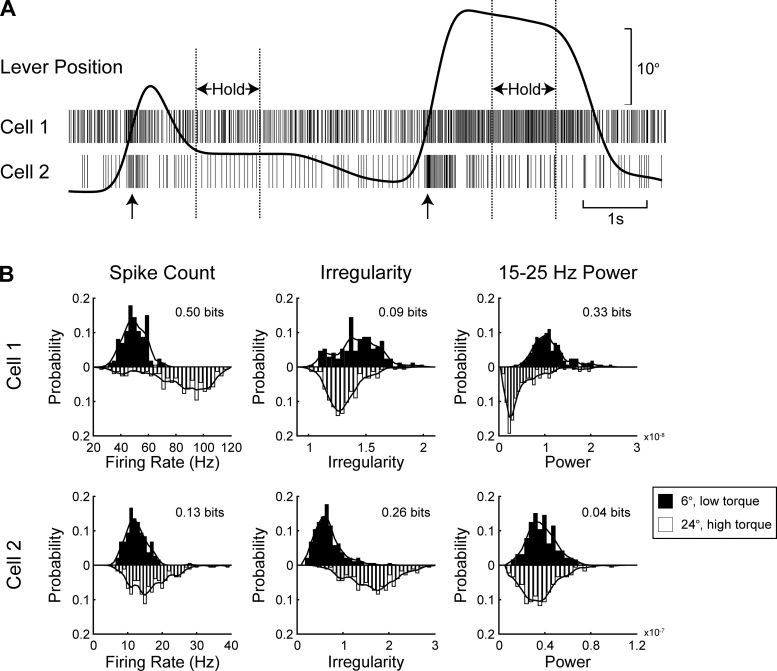
Example recordings and outline of analysis. *A*: spike times from two area 2 cells together with lever position for two successive trials (6°, low-torque followed by 24°, high-torque conditions). Dotted lines show hold periods used in subsequent analysis. Arrows indicate times of peak velocity. *B*: histograms of spike count, irregularity and 15–25 Hz power for the 2 cells shown in *A*. Filled bins show histograms for the 6°, low-torque trials, and open bins show histograms for the 24°, high-torque trials. Lines overlying histograms show the smoothed estimates of the probability density function. Information value for the 8-condition task is shown in *top right* corner of each plot (see methods).

We began by finding the time of peak velocity of the finger displacement at the start of each successful trial. Subsequent analysis used a 1.024-s-long time window, starting 0.977 s after the peak velocity (see Fig. 1*A*). This period, and the use of peak velocity as the alignment event, was chosen to yield the most consistent and stable traces of finger displacement. For each cell, the spikes occurring during this window for each trial were extracted and three parameters of firing measured. The number of spikes in the window, *N*_spike_, yielded the spike count. Spike irregularity IR was calculated according to Davies et al. (2006):

(1) $$IR=\sum_{i=1}^{i=N-1}|\log\left(I_{i+1}/I_{i}\right)|,$$

where *I*_*i*_ denotes the *i*th ISI (*I* = 1…*N*), |·| denotes absolute value, and log is the natural logarithm. Low values of IR indicate a more regular spike train. Finally, since our previous work has shown that ∼20-Hz oscillations are present in the areas recorded from and are strongest during the hold phase of the task, we estimated the spectral power of the spike train in the range 15–25 Hz. The spike train was converted to a waveform sampled at 1 kHz by counting spikes in 1-ms nonoverlapping bins. If the 1,024-point discrete Fourier transform of this process is represented as *X*_*i*_ (where *i* indexes the frequency bin), the 15–25 Hz power was measured as

(2) $$Power=\frac{1}{N_{spike}}\sum_{i=15}^{i=25}X_{i}X_{i}^{*}.$$

Note the normalization by the spike count. Because each 1-ms bin of the spike train time series holds either 0 or 1 spike, the sum of the bin counts equals the sum of the squares of the bin count, which is equal to the total power. Normalizing by the total spike count *N*_spike_ is thus equivalent to measuring 15–25 Hz power as a proportion of the total at all frequencies. With this normalization, changes in rate will not automatically lead to power changes. All three measures were made for each trial separately.

#### Information about task condition: single parameter.

The task information available from each of the three parameters was calculated as follows. For each of the eight task conditions, a histogram was formed of the distribution of the parameter across trials. For spike count, discrete bins with width one count were used. For spike irregularity and spike power, there were 40 equally spaced bins, stretching from the minimum to the maximum value of that parameter measured in that cell. The histograms were then smoothed with a Gaussian kernel, width σ = 1 bin. This gave the probability distribution of the neural response given task condition, *P*(R|C). By combining all trials together, ignoring the task condition, we obtained the overall probability distribution of the neural response, *P*(R). Example histograms for the 6°, low-torque and 24°, high-torque conditions for each parameter are shown in Fig. 1*B* for two cells recorded from cortical area 2.

The entropy *H* of these probability distributions and the information *I* were calculated using the following formulas (Ash 1990):

(3) $$\begin{array}{l} H\left(R\right)=\overset{J}{\underset{j=1}{\sum }}-P\left(R_{j}\right)\log_{2}\;P\left(R_{j}\right)\\ H\left(R|C\right)=\overset{8}{\underset{i=1}{\sum }}P(C_{i})\overset{J}{\underset{j=1}{\sum }}-P\left(R_{j}|C_{i}\right)\log_{2}\;P\left(R_{j}|C_{i}\right)\\ I=H(R)-H(R|C). \end{array}$$

Because of limited sampling, there is a bias in this entropy calculation which affects *H*(R|C) more than *H*(R), leading to an upward bias in the information (Treves and Panzeri 1995). This was corrected for by using the quadratic expansion method (Strong et al. 1998). The biased entropy *H*_EST_ was assumed to be related to the actual entropy *H*_ACT_ by

(4) $$H_{EST}=H_{ACT}+\frac{a}{N}+\frac{b}{N^{2}},$$

where *N* is the number of trials used to calculate *H*_EST_, and *a* and *b* are constants. By dividing the available trials into two and four sections and recalculating the entropy, *H*_EST_ was found for *N*, *N*/2, and *N*/4 trials. *H*_ACT_ and the other parameters were then determined by quadratic fitting.

Whether information was significantly different from zero was tested by randomly shuffling the parameter values across task condition and recalculating the information as above. This was repeated 1,000 times; the 95th centile of information in shuffled data provided the significance level (*P* < 0.05).

To test whether a neuron coded for torque or displacement individually, a similar analysis was carried out, but now trials were divided only according to displacement (4 conditions) or torque (2 conditions).

#### Joint information about task condition: two parameters.

When two parameters individually carried significant information about the task, we then asked whether simultaneous knowledge of both parameters coded significantly more or less information than expected from summation of the individual information values, which would correspond to independent coding. This analysis started by constructing two-dimensional histograms for *P*(R) and *P*(R|C), which yielded the joint information, using the same bin sizes as for the single-parameter analysis. Information bias depends very sensitively on the number of bins and the sparseness with which they are filled, and would therefore differ considerably for these two-dimensional histograms compared with the one-dimensional histograms used to estimate information from single variables. Although we did correct for bias as described above, small errors in this process could lead to systematic errors in the comparison of the joint information with the sum of the individual information values. We therefore developed a shuffling approach that compared joint information values from two-dimensional histograms only with those also determined from two-dimensional histograms.

**Fig. 2.**
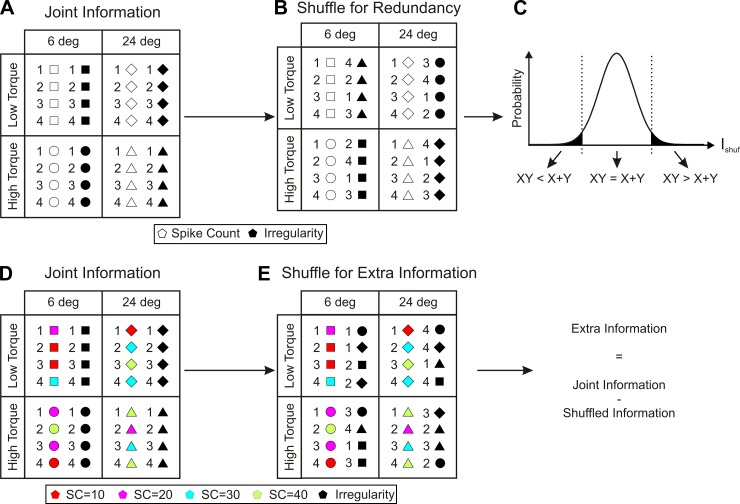
Shuffling method for joint information. Symbols represent spike count (open) and irregularity (filled) values on different trials. Numbers represent the order of trials within each condition (i.e., 1 represents 1st trial of that condition). Different symbol shapes represent different task conditions (i.e., squares represent 6° position, low-torque condition). *A*: calculation of joint information used original data, with spike count and irregularity values paired as measured experimentally. *B*: to calculate redundancy, irregularity values were first shuffled en masse between conditions; the order was then shuffled within a condition. The joint information was then calculated. This destroyed any association between the variables and produced a prediction of the information expected if coding was independent. *C*: schematic representation of the probability distribution of information from data in *B* after shuffling procedure was repeated 1,000 times. Shaded areas represent 2.5% tails. If the original joint information (*A*) falls into one of these shaded areas, it is said to be either redundant (*XY* significantly less than *X* + *Y*, where *XY* is the joint information and *X* and *Y* are the individual information values) or synergistic (*XY* significantly greater than *X* + *Y*). *D*: for calculation of extra information, trials were first classified according to the spike count measure, represented as different colors of spike count symbols. *E*: shuffling of irregularity values was performed within the same values of spike count. Information was estimated from the shuffled data and compared with experimental data (*A*) to determine extra information coded by irregularity. SC, spike count.

The aim of shuffling was to instantiate the null hypothesis that information coding was independent. Information determined from the actual experimental data set could then be compared with that from the shuffled data sets and any statistical deviation determined. Two possible connections between coding by rate and irregularity needed to be destroyed by this shuffling (Pola et al. 2003). The first was any correlation between the mean responses in different task conditions. This was achieved by shuffling different task conditions en masse. Figure 2, *A* and *B*, illustrates this schematically. For example, in the original data, the 6°, low-torque trials are shown as squares. Following the first part of the shuffling, the irregularity values from these trials have all been moved to 6°, high torque. This is effectively just a relabeling of task conditions. In the second stage of the shuffling, irregularity values were shuffled within a given task condition (note the reordered trial numbers). This destroyed any trial-to-trial correlation between rate and irregularity. Importantly, information measured from irregularity alone based on these shuffled values would be the same as from the original experimental data set; the single variable information has been preserved.

Information from the joint histogram was measured from the shuffled data set, and then the shuffle process was repeated 1,000 times. The distribution of shuffled information was then compiled (Fig. 2*C*). If the actual information value fell within the left tail of the distribution, this corresponded to redundancy: significantly less information could be obtained from the parameter pair together than expected from the sum of what we would learn by knowing each of them alone. By contrast, if the actual information value fell in the right tail of the distribution, this corresponded to synergy: we learned more from knowing both parameters simultaneously than we would expect from a simple summation of what could be learned from each alone. Finally, if the actual information value fell within the middle part of the shuffle distribution, this corresponded to independence: we had no statistical evidence that information from the two parameters did anything other than add linearly.

Although two measures may be redundant, knowledge of both may still carry extra information over and above that provided by one measure alone. To calculate the extra information carried by one measure when another was known, a different shuffling procedure was used, illustrated in Fig. 2, *D* and *E*. We first identified all trials that had a given value of spike count; these have been color coded in the schematic of Fig. 2*D* so that, for example, all trials with red spike count markers had a spike count value of 10. Irregularity values were then shuffled between these trials. Careful inspection of Fig. 2*E* shows that irregularity values have been moved while respecting their spike count classification. This procedure preserves any potential correlation between spike count and irregularity but destroys any additional information that might be coded by the combination of the two measures compared with spike count alone. As for independence, a distribution of the information found from 1,000 shuffles was found and compared with the experimental value. If the experimental value fell within the right tail of this distribution, it provided statistical evidence for the encoding of extra information by irregularity.

#### Neural model.

To investigate how irregularity and rate could vary independently, we carried out some simple numerical simulations. These used a variant of the model first described by Matthews (1996) and used subsequently in our own work on postspike membrane potential trajectories (Wetmore and Baker 2004; Witham and Baker 2007). Following a spike, the membrane potential *V* was assumed to traverse the following trajectory:

(5) $$V(t)=A-3e^{-\frac{t}{20}}+He^{\frac{-{\left(t-50\right)}^{2}}{10^{2}}}+\varepsilon (t),$$

where *t* is time (in ms) following the spike. This is an exponential decay from a hyperpolarized level toward the spike threshold (arbitrarily taken as *V* = 0), plus a depolarizing peak of height *H* and a constant depolarization *A*. Synaptic noise ε(*t*) was simulated as Gaussian white noise, filtered through a membrane time constant of 12 ms. In this class of neural models, the noise standard deviation (SD) is usually set to 1, and *V* is then expressed in “noise units.” For the present purposes, we wanted to test the impact of changes in input noise and hence ran simulations with either the standard SD of 1 or a higher SD of 2. The model was simulated with a time step of 1 ms. In each iteration, *V*(*t*) was updated according to *Eq. 5*; if *V*(*t*) > 0, a spike was taken to have occurred. The spike time within the simulation was then noted and the trajectory reset (*t* = 0).

Simulations of this model were run with either *H* = 0 or *H* = 0.6, to simulate a cell with exponential or peaked postspike membrane trajectory, respectively. Different simulation runs varied the parameter *A* to alter firing rates over the approximate range 1–80 Hz (*A* = −1…3 for *H* = 0; *A* = −2.5…1.5 for *H* = 0.6). After a given simulation had run for 500 s, we calculated the mean firing rate and the irregularity of discharge. The parameters in *Eq. 4* and values of *h* were chosen to be reasonable in the light of our previous experimental measurements (Wetmore and Baker 2004; Witham and Baker 2007).

## RESULTS

**Fig. 3.**
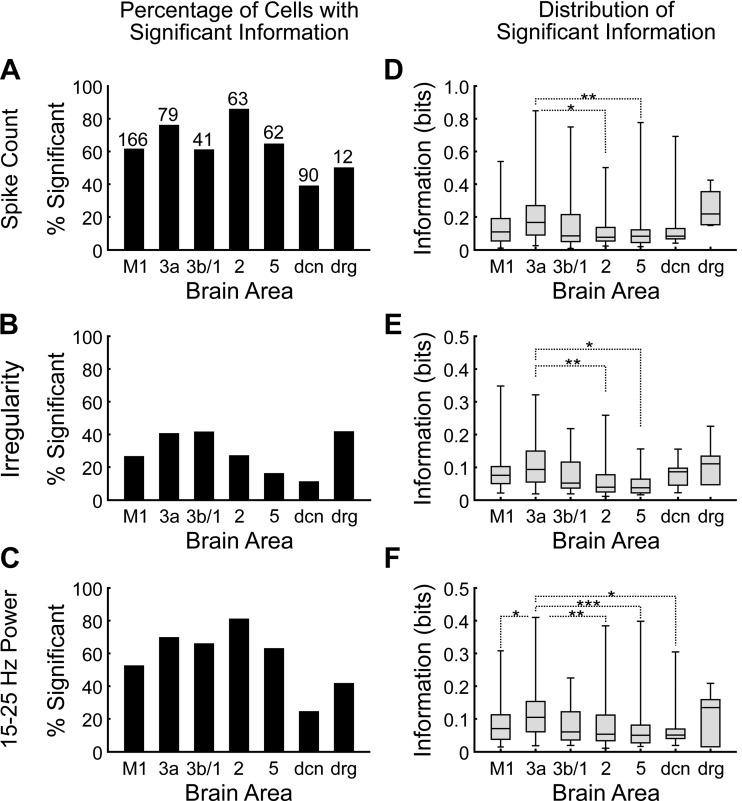
Information values for the 8-condition task. *A–C*: percentage of cells with significant task information measured using spike count (*A*), irregularity (*B*), and spike power (*C*) for 7 different recording areas (dcn, deep cerebellar nuclei; drg, dorsal root ganglion). Total numbers of cells used for analysis are indicated above the bars in *A*. *D–F*: distribution of significant information from spike count (*D*), irregularity (*E*), and spike power (*F*). For each area, the minimum, lower quartile, median, upper quartile, and maximum are plotted. Dotted lines show pairs of areas for which there was a significant difference in information (Kruskal-Wallis test with Bonferroni correction for multiple comparisons: *P < 0.05; **P < 0.01; ***P < 0.001). D

Cells were recorded from 8 different areas of the nervous system in 2 monkeys; a total of 501 cells showed significant modulation in firing rate with the task and were therefore used for further analysis in this report. Cells from area 3b and area 1 were grouped together for analysis. The distribution of cells across the areas is shown in Fig. 3*A* (total number of cells indicated above each bar). For each cell, information about task condition was calculated using spike count, irregularity, and 15- to 25-Hz spectral power; results are shown in Fig. 3.

All areas had cells with significant task information (see methodsfor details of significance calculation) in their spike counts (Fig. 3*A*). Area 2 had a significantly larger proportion of significant cells than any other area except for area 3a (χ^2^ test, *P* < 0.05, pairwise comparison with Bonferroni correction for multiple comparisons). Area 3a had significantly higher spike count information than area 2 and area 5 (Fig. 3*D*; Kruskal-Wallis test, *P* < 0.05 with Bonferroni correction for multiple comparisons). In all areas, a smaller percentage of cells had significant task information in their irregularity (Fig. 3*B*). Area 3a, area 3b/1, and the DRG had the highest proportion of cells coding task information significantly in irregularity. Area 3a had significantly higher irregularity information than area 2 and area 5 (Fig. 3*E*; Kruskal-Wallis test, *P* < 0.05 with Bonferroni correction for multiple comparisons). All areas had cells with significant information in their 15–25 Hz power (Fig. 3*C*). The values of information were similar to those for irregularity, and again, area 3a had a significantly higher level of information (Fig. 3*F*).

### 

#### Displacement and torque information.

**Fig. 4.**
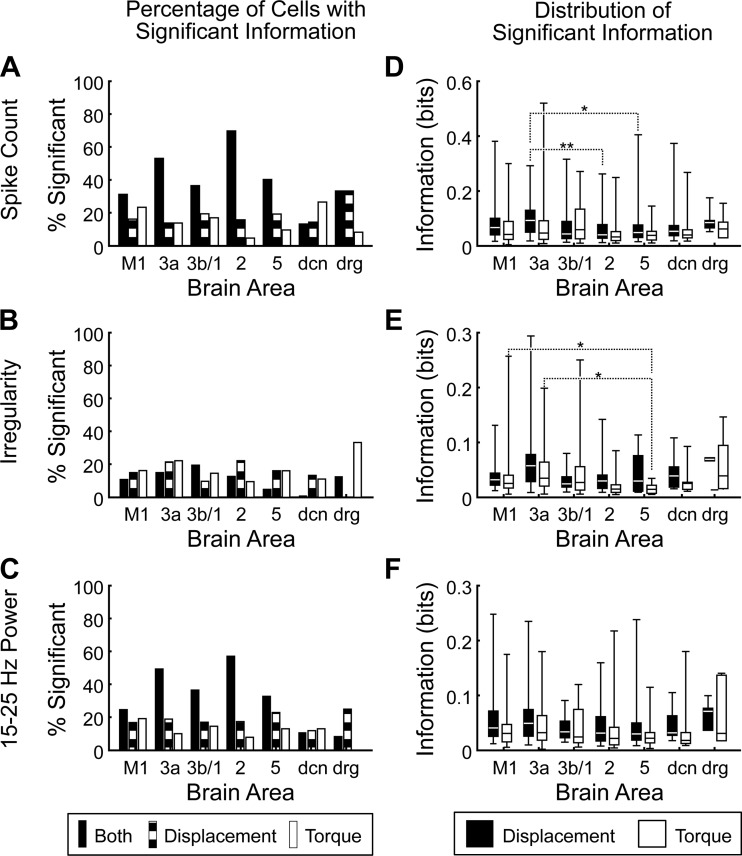
Information about displacement and torque. *A–C*: percentage of cells with significant information about displacement (striped bars), torque (open bars), or both displacement and torque (solid bars) measured using spike count (*A*), irregularity (*B*), and spike power (*C*). *D–F*: distribution of significant information about displacement (solid bars) and torque (open bars) measured using spike count (*D*), irregularity (*E*), and spike power (*F*). For each area, the minimum, lower quartile, median, upper quartile, and maximum are plotted. Dotted lines show pairs of areas for which there was a significant difference in information (Kruskal-Wallis test with Bonferroni correction for multiple comparisons: *P < 0.05; **P < 0.01).

Information about displacement was calculated by categorizing trials based only on the target displacement, ignoring opposing torque; because there were four possible displacements, the maximum information was 2 bits. A similar analysis was calculated for torque; because there were two possible torques, maximum information in this case was 1 bit. Figure 4, *A–C*, shows the proportion of cells coding significant information about displacement or torque alone and those that coded both. In all cortical areas for both spike count and spectral power, the most common result was for cells to code both position and torque (Fig. 4, *A* and *C*); for cells with significant spike count or power information, approximately half (52.4% for spike count and 48.9% for spike power) carried both position and torque information. By contrast, irregularity more commonly coded either position or torque alone (Fig. 4*B*). Only 24.6% of cells with significant irregularity information carried both position and torque information; the remaining cells were split fairly evenly between position (38.8%) and torque (36.5%). For all three measures there were no significant differences between the number of position only cells and the number of torque only cells (χ^2^ test with Bonferroni correction for multiple comparisons, *P* > 0.05).

In general, information about displacement was higher than information about torque for all three measures, as might be expected since there was more potential information about displacement than about torque (significant for all 3 measures: *P* < 0.001, Wilcoxon rank sum test). For those cells which coded significant information about both behavioral variables, there was only a small correlation between position and torque information (*r*^2^ values 0.099 for spike count, 0.169 for spike irregularity, and 0.127 for 15–25 Hz power).

#### Relationship between information carried by spike count, irregularity, and 15–25 Hz power.

Irregularity and 15–25 Hz power both coded significant information about this task, which may suggest the use of temporal coding. However, it is important to determine whether this is independent of information carried by firing rate. We used a novel shuffling method (see methods and Fig. 2) to examine this. Coding was said to be redundant if the joint information was significantly less than the sum of the information obtained from the two measures alone (*XY* < *X* + *Y*). Coding was synergistic if joint information was significantly greater than the sum of the individual information (*XY* > *X* + *Y*). Coding was independent if there was no statistical evidence that the joint information was other than the sum of the individual values. These comparisons were made first between each pair of measures from a single cell, using only cells for which there was significant information carried by both measures individually. We then additionally carried out the same analysis for information coded by the same measure between two simultaneously recorded neurons.

**Fig. 5.**
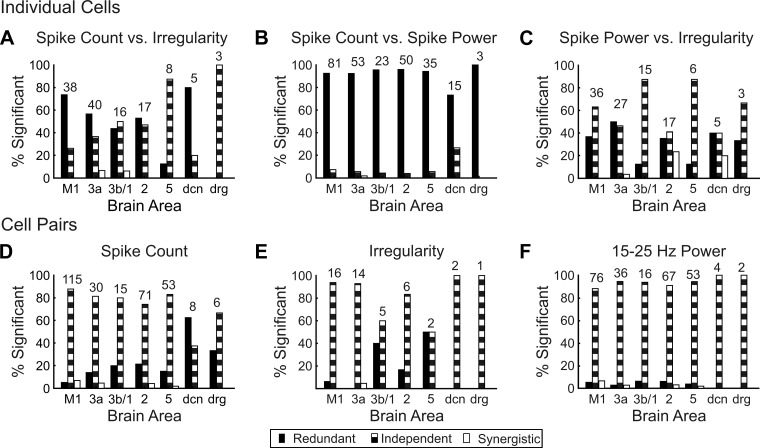
Redundancy and independence in spike count, irregularity, and spike power information. *A–C*: percentage of cells with redundant (solid bars), independent (striped bars), and synergistic (open bars) interactions between spike count and irregularity information (*A*), spike count and spike power information (*B*), and spike power and irregularity information (*C*). *D–F*: percentage of cell pairs with redundant (solid bars), independent (striped bars), and synergistic (open bars) interactions within spike count information (*A*), irregularity information (*B*), and spike power information (*C*). Total numbers of cells used for analysis are indicated above bars for each area.

The results of the analysis performed on pairs of measures from the same cell are shown in Fig. 5, *A–C*. Spike count and 15–25 Hz power (Fig. 5*B*) were most often redundant, indicating that they conveyed at least some of the same information (total across 260 cells from all areas: 92.7% redundant, 6.9% independent, 0.4% synergistic). By contrast, irregularity was most often independent of both spike count (Fig. 5*A*; total across 114 cells from all areas: 56.4%, redundant, 41.0% independent, 2.6% synergistic) and 15–25 Hz power (Fig. 5*C*; total across 109 cells from all areas: 34.8% redundant, 60.0% independent, 5.2% synergistic).

We also used tested for redundancy in information coded by the same measure in two simultaneously recorded cells; results are shown in Fig. 5, *D–F*. Note that the number of cell pairs that both coded significant information with irregularity was much smaller than for the other two measures (see numbers above each set of bars); Fig. 5*E* is therefore based on a very small database, and the results should be treated with caution. For the majority of cell pairs, irrespective of area or measure, information was represented independently (62, 524, and 24 of 612 pairs across all areas and measures were redundant, independent, and synergistic, respectively). A small number of cells pairs were recorded from the same electrode and separated on the basis of differences in their spike waveforms. Because these neurons are likely to be very close neighbors, we might expect substantial shared input and greater redundancy in their coding. However, these cell pairs showed the same pattern as the rest of the cell pairs, with the most common result being independence (4, 26, and 1 of 31 pairs across all areas and measures were redundant, independent, and synergistic, respectively).

#### Extra information in irregularity and spike power.

**Fig. 6.**
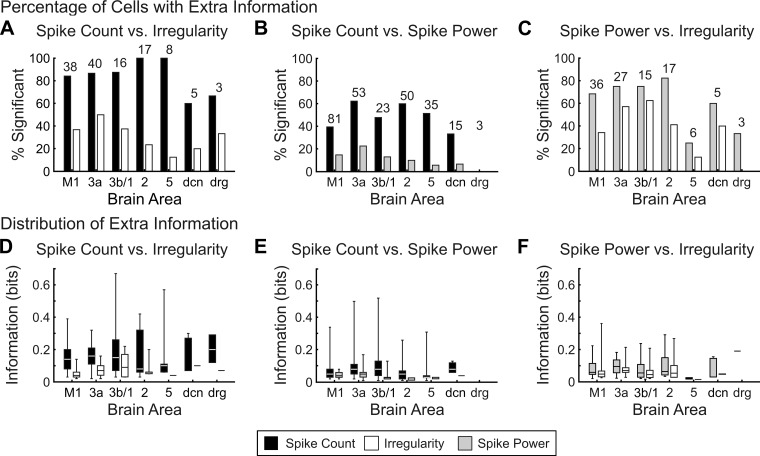
Extra information from interactions between spike count, irregularity, and spike power. *A–C*: percentage of cells with significant extra spike count information (solid bars), extra irregularity information (open bars), and extra spike power information (shaded bars) for spike count and irregularity interactions (*A*), spike count and spike power interactions (*B*), and spike power and irregularity interactions (*C*). Total numbers of cells used for analysis are indicated above bars for each area. *D–F*: distribution of significant extra spike count information (solid bars), extra irregularity information (open bars), and extra spike power information (shaded bars) for spike count and irregularity interactions (*D*), spike count and spike power interactions (*E*), and spike power and irregularity interactions (*F*). For each area, the minimum, lower quartile, median, upper quartile, and maximum are plotted.

Even if two measures are redundant, it may still be the case that knowledge of both provides more information than either one alone. We therefore used a modified version of the shuffling analysis (see methods) to determine whether one measure provided significant additional information, given that another measure was already known. The percentage of cells with significant extra information for each of the two measures in a pairwise comparison is shown in Fig. 6, *A–C*. In the great majority of neurons, spike count coded additional information compared with irregularity (solid bars in Fig. 6*A*, 87.2% of cells) and 15–25 Hz power (solid bars in Fig. 6*B*, 49.6% of cells). By contrast, it was less common for either measure related to temporal coding to contain extra information relative to spike count: 35.9% of cells for irregularity (open bars in Fig. 6*A*) and 13.5% of cels for 15–25 Hz power (shaded bars in Fig. 6*B*). There was evidence that irregularity and 15–25 Hz power coded somewhat different information: significant extra information in irregularity relative to power for 42.6% of cells, and in power relative to irregularity for 68.7% of cells.

For cells where significant extra information was carried by one measure compared with another, we then calculated the size of this information gain; this is illustrated in Fig. 6, *D–F*. The amount of extra information carried by spike power and irregularity on top of spike count was similar. There were no significant differences between the different areas (probably partly due to the small number of cells analyzed; Kruskal-Wallis test, *P* > 0.05 with Bonferroni correction for multiple comparisons). The extra information carried by all three measures was lower than the information calculated for the individual measures (spike count: median 0.078 bits extra vs. 0.171 bits individual; irregularity: median 0.055 bits extra vs. 0.085 bits individual; 15–25 Hz power: median 0.057 bits extra vs. 0.122 bits; all significant: *P* < 0.001, Wilcoxon signed rank test with matched pairs).

#### Comparison with peaks in afterhyperpolarization potentials.

One possible mechanism underlying regular firing in cells is a depolarizing peak in the trajectory of the membrane potential following a spike. In a previous paper (Witham and Baker 2007), we used a statistical analysis of ISIs to show that a significant proportion of cells in sensorimotor cortex and the DCN have peaks in their postspike trajectories, which could produce more regular firing. We investigated whether there was any relationship between a peaked postspike trajectory and the amount of information carried by irregularity. First, we found that the median irregularity of cells with peaked trajectories was lower than that of cells without peaks (median IR 0.79 for 251 cells with peaks compared with 0.87 for 250 cells without; *P* < 0.05, Wilcoxon rank sum test). We then looked at the information carried by irregularity and found that a significantly larger proportion of cells with peaked trajectories had significant information in irregularity (80/251 cells with peak compared with 43/250 cells without; *P* < 0.01, χ^2^ test). Cells with peaked trajectories also had significantly larger information carried by irregularity than cells without peaks (median information 0.0286 compared with 0.0212 bits; *P* < 0.05, Wilcoxon rank sum test). A similar analysis performed for spike count and spike power found no significant differences between cells with peaked trajectories and those without.

#### Simulating the effect of different postspike membrane potential trajectories on coding using spike irregularity.

**Fig. 7.**
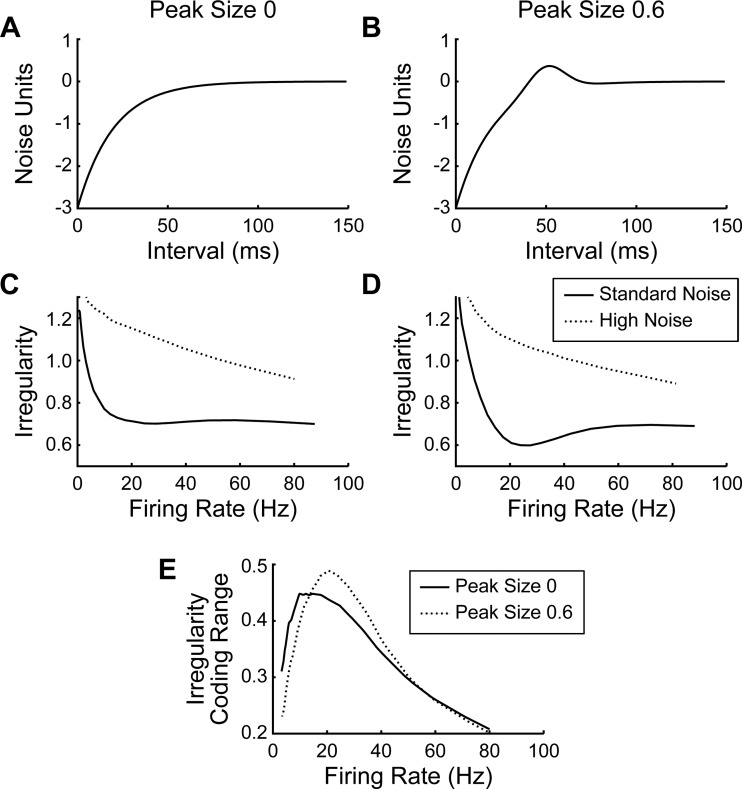
Effects of postspike membrane trajectory on irregularity assessed using a computational model. *A* and *B*: postspike trajectories, tested in the model, which either rose monotonically toward threshold (*A*) or incorporated a peak (*B*). *C* and *D*: variation of spike irregularity with firing rate, for simulations run with the postspike trajectories shown in *A* and *B*. Solid line shows results for models with noise SD of 1 unit; dotted line shows results for noise SD of 2 units. *E*: difference in irregularity between high noise and standard noise simulations for models using the monotonically rising (solid line) or peaked (dotted line) postspike trajectories.

We were interested in how the shape of the postspike membrane trajectory altered the ability of a neuron to modulate its spiking variability. We therefore used a simple numerical simulation of a neuron responding to noisy inputs; details of the model are given in methods. Following a spike, the membrane potential was assumed to traverse a fixed trajectory from a hyperpolarized state back toward spiking threshold. This initial part of the postspike trajectory effectively models the relative refractory period, because the probability of spiking is reduced during the hyperpolarized region immediately after a spike. Two trajectories were examined: one that rose monotonically (Fig. 7*A*) and the other that included a peak (Fig. 7*B*). The size and timing of this peak was chosen to match approximately some of the more strongly peaked trajectories seen in the experimental data (Witham and Baker 2007).

By running this model for different levels of constant depolarization (parameter *A* in *Eq. 5*; see methods), we produced simulations where the mean firing rate varied from 1 to 80 Hz. We could then look at how irregularity varied with firing rate for the different shapes of membrane potential trajectory. Figure 7, *C* and *D*, plots how the spike irregularity in these simulations varied with firing rate for the monotonic and peaked trajectory, respectively. The solid lines illustrate results when the SD of the synaptic noise input to the cell was set to unity, which is the usual value in such “distance to threshold trajectory” models. For the monotonic trajectory, irregularity decreased with increasing firing rate up to ∼20 Hz, but then was fairly constant for higher rates. This reflects the impact of the relative refractory period, which causes spike trains to become more regular at higher rates (Berry and Meister 1998). For the peaked trajectory, there was an additional reduction in irregularity around 20 Hz, reflecting an increased propensity of the cell to fire at the time of the peak (50 ms).

These changes in irregularity occur in an obligate way with changes in rate and as such could not produce additional coding of information via irregularity. However, independent changes in irregularity could occur if the level of synaptic noise in the neuron was altered, as might occur if there were changes in the relative balance of excitatory and inhibitory drive (Destexhe 2010).To examine this, we reran the simulations with a level of noise SD twice the usual value; these results are shown as dotted lines in Fig. 7, *C* and *D*. For both trajectories, the firing irregularity was considerably increased and now continued to depend on rate up to the maximum rate examined.

The plots of Fig. 7, *C* and *D*, illustrate the range of firing irregularity that each cell model can support at a given firing rate, just by changing the synaptic input noise over the tested range of SD. Figure 7*E* plots the difference between the two curves of Fig. 7, *C* and *D*, corresponding to the maximum change in irregularity that each cell can produce at a given firing rate. This will limit the extent to which parameters can be coded using changes in irregularity by that cell. For firing rates from 14.4 to 56.0 Hz, the model with the peaked distance-to-threshold trajectory showed a larger range of irregularity.

## DISCUSSION

In this report, we examined coding of displacement and torque in a finger movement task across a range of cortical and subcortical regions. Significant information about these task parameters was encoded not only by spike count but also by irregularity and 15- to 25-Hz spectral power, which are sensitive to the temporal structure of the spike train. We showed that the information carried by temporal and rate codes was frequently complementary: more could be learned about task condition from knowledge of two measures than from one alone.

Two analyses were used to examine how information in pairs of measures interacted. We first tested whether total information added linearly. In all instances, synergy was rare, and there was little evidence that knowing two measures could yield significantly greater information than expected from the linear sum. Spike count and the 15–25 Hz power in spike discharge often showed redundancy. We have previously shown that spike rate and LFP spectral power show different time courses in coding for digit displacement. On average, peak information in rate occurs earlier than for LFP, but there is considerable heterogeneity, and a significant minority (30%) of cells code peak information later than seen in LFP recorded at the same site (Witham and Baker 2012). It appears that beta-band oscillations and neural discharge rate cooperate to evolve a representation of task parameters during the steady hold phase; it is therefore perhaps not surprising that there should be extensive redundancy in representation between spike count and 15–25 Hz power.

Our previous work studied coding in LFP oscillations, which reflect synchronous activity in a local network. In the present study, we measured 15- to 25-Hz spectral power in neural spiking. This is a more focused signal and could be modulated by several distinct mechanisms. Increases in network oscillations will lead to oscillatory subthreshold membrane potential oscillations in all cells forming part of the network. Single-neuron discharge is coherent with LFP oscillations, confirming that the power spectrum of cell spiking does partially reflect local network activity (Baker et al. 2003). Additionally, spike 15–25 Hz power will reflect the tendency to rhythmic firing of the single cell. In sensorimotor cortex we have previously shown that some cells have peaked postspike trajectories, leading to a tendency for rhythmic firing (Wetmore and Baker 2004; Witham and Baker 2007). This is unrelated to the presence or absence of synchronous network oscillations (Witham and Baker 2007). In this work we saw that irregularity and 15–25 Hz power were often independent (Fig. 5*C*). This suggests that 15–25 Hz power in these single-neuron spike trains was most closely related to synchronized local network oscillations, rather than to intrinsic properties of the cells. Irregularity also often showed coding independent from spike count, suggesting that this reflects a distinctly different representation, which may arise via separate mechanisms from those yielding rate and oscillatory coding.

The second analysis that we carried out looked at whether one measure carried any extra information beyond what was given by another measure. The difference with the use of this approach and how it looked at redundancy/independence can best be illustrated by an example. Suppose spike count and irregularity each separately code 0.1 bits of information, but together they code 0.15 bits. This represents redundancy, because the combined coding is smaller than the expected linear sum of 0.2 bits. On the other hand, adding a second measure does improve the coding over use of a single measure (by 0.05 bits), so there is extra information. In comparing the results on extra information and independence, it is important to note a subtle difference in the statistical question asked. When we test for redundancy, independence, or synergy, independence is the null hypothesis. Rejecting this null hypothesis requires an effect large enough to rise out of the statistical noise. It is likely therefore that some of the cells were classed as “independent” simply because there was insufficient data to render a weak effect significant. By contrast, when we test for extra information, the null hypothesis is that there is zero extra information; we are likely to see false negatives caused by insufficient data. This explains why the number of cells showing extra information is not equal depending on which way the analysis is conducted. For example, 84.2% of M1 cells have significant extra information in spike count compared with irregularity, but only 36.8% of M1 cells have significant extra information in irregularity compared with spike count (Fig. 6*A*). The information carried in spike count is usually higher than that in irregularity (Fig. 3, *D* and *E*; Fig. 5*D*), making extra information in spike count more likely to be detected statistically.

When we examined coding by pairs of cells, the most common result was that coding was independent between pairs (Fig. 5, *D–F*). Again, this may partly reflect statistical thresholding and a failure to detect weak redundancy given limited data. It does, however, imply that redundancy is not extensive, reflecting largely uncorrelated signal and noise components of the cell discharge. This is to be expected on the basis of previous work: cell-cell coherence values, although detectable, are low, and noise components of discharge unrelated to LFP oscillations also appear largely uncorrelated (Baker et al. 2003).

### 

#### Coding by irregularity.

One interpretation of the temporal coding hypothesis is that neurons generate precisely timed sequences of spikes and that these sequences are exactly reproduced if the same stimulus or behavioral condition is replicated (Abeles 1991). However, an alternative possibility is to see temporal coding in more statistical terms: rather than precise interval patterns being replicated from one trial to the next, coding would then be via average differences in temporal features of the spike train, such as the proportion of synchronous spikes or the spectral power in a given band. Coding by spike train irregularity is an example of one such statistical temporal code.

As noted above, in many cases information encoded by spike irregularity was independent from that in spike count or 15–25 Hz power. Furthermore, the nature of what was encoded appeared different, with irregularity more often coding either torque or displacement, whereas in the majority of cells spike rate and 15–25 Hz power coded features of both task parameters. Previous experimental work has shown that parallel increases in excitatory and inhibitory inputs to a neuron can increase firing irregularity without necessarily changing firing rate (Gauck and Jaeger 2000). In the present study, we simulated neurons responding to noisy inputs (Fig. 7) and showed that increasing the input noise increased irregularity of output spiking, confirming previous computational modeling studies (Feng and Brown 1998; Salinas and Sejnowski 2002; Stein 1965). We also compared how the size of this effect differed between model neurons with peaked and monotonically rising postspike trajectories. Within a range that encompassed most of the naturally occurring firing rates, the model cell with peaked trajectory could modulate its output irregularity more following changes in input noise. In our experimental data set, we showed that cells with significant peaks in their postspike trajectories were more likely to encode significant task information in irregularity, and the amount of information encoded was higher, compared with cells that had monotonic membrane potential trajectories.

Taking modeling and experimental results together, it seems likely that coding of task parameters by irregularity reflected an interaction of changes in synaptic noise with the intrinsic membrane properties of the cell. Although this is a plausible explanation for the cause of the observed encoding, the question remains whether changes in irregularity can be read by downstream neurons, an essential prerequisite if this is genuinely a neural code. In this regard, Tsodyks and Markram (1997) have reported that cortical pyramidal neurons can be sensitive to different patterns of input depending on the rate of synaptic depression. This parameter shows considerable heterogeneity between cells and could be subject to modification both by spike timing-dependent plasticity and by neuromodulators such as acetylcholine. This may provide a mechanism by which downstream neurons can be “tuned” to varying levels of spiking irregularity.

#### Conclusions.

In the present work, we have provided evidence that both rate and temporal codes can represent task parameters in the primate sensorimotor system. The same general pattern of results was seen both in the cerebral cortex, DCN, and peripheral afferents of the DRG. The precarious nature of our DRG recordings prevented reliable determination of the receptive field of the afferents, so we cannot be sure whether cutaneous or deep receptors were involved. However, it has previously been shown that regularity of muscle spindle afferent discharge depends on the activation of gamma innervation (Matthews and Stein 1969) and that afferent fibers can encode oscillations seen in muscle activity (Baker et al. 2006); it is thus not surprising that if we can find significant temporal coding in central neurons, this is also reflected in the periphery. Our results mirror similar findings in sensory systems for “multiplexed” representation at both short and long timescales (Panzeri et al. 2010).

As well as having considerable interest for our understanding of neural computation in motor control, these results may have practical implications for the field of brain-machine interfaces (BMI), which seek to control external effectors from multiple neural recordings. Much effort is currently being expended to enhance the performance of these systems, including the design of more sophisticated decoding algorithms and improvements to chronic neural recording. However, almost all decoders begin from neural firing rate estimated in brief time windows (Collinger et al. 2013; Hochberg et al. 2012) or spectral power and phase of field recordings such as electrocorticogram (Pistohl et al. 2013) or LFP (Flint et al. 2013). Spiking irregularity can be rapidly calculated online using only a few ISIs and seems to code a source of information distinct from that represented in rate or power. We suggest that BMI applications may benefit from incorporation into decoding algorithms alongside more established approaches.

## GRANTS

This work was funded by the Wellcome Trust.

## DISCLOSURES

No conflicts of interest, financial or otherwise, are declared by the authors.

## AUTHOR CONTRIBUTIONS

C.L.W. and S.N.B. conception and design of research; C.L.W. and S.N.B. performed experiments; C.L.W. and S.N.B. analyzed data; C.L.W. and S.N.B. interpreted results of experiments; C.L.W. and S.N.B. prepared figures; C.L.W. and S.N.B. drafted manuscript; C.L.W. and S.N.B. edited and revised manuscript; C.L.W. and S.N.B. approved final version of manuscript.
